# A Massive Sliding Inguinal Hernia Containing the Entire Right Colon With Caecal Perforation: A Rare Surgical Challenge

**DOI:** 10.7759/cureus.93330

**Published:** 2025-09-27

**Authors:** Marwa S Ahmed, Tawfiq Hamati, Farooq Dar, Gassan Salih

**Affiliations:** 1 General and Colorectal Surgery, Barnsley Hospital NHS Foundation Trust, Barnsley, GBR; 2 General Surgery, Barnsley Hospital NHS Foundation Trust, Barnsley, GBR; 3 Urology, Barnsley Hospital NHS Foundation Trust, Barnsley, GBR

**Keywords:** abscess, case report, hernia, laparotomy, swelling

## Abstract

Hernias of the abdominal wall result from an area of weakness or defect in the abdominal wall, specifically in the area between the ribs and the hips. It can appear anywhere across the abdominal wall. The most common type of hernia is inguinal, which affects the groove between the abdomen and thigh. We discuss the case of a 75-year-old male who was admitted to the hospital after presenting with a large right-sided inguinoscrotal hernia, which had been gradually growing for the last three years. The patient had acute pain, hard swelling, and redness for the past three days. The general assessment revealed a large irreducible hernia, which was tender in the area of erythema. An emergency CT of the abdomen and pelvis (CTAP) demonstrated a perforated ascending colon with the formation of abscesses within the hernia.

The management involved an exploratory laparotomy, which showed evidence of a strangulated hernia; there was extensive ischaemia and congestion of the terminal ileum, cecum, appendix, ascending colon, and omentum. The surgical interventions included right hemicolectomy with ileostomy, drainage of the abscess in the abdominal wall, and hydrocelectomy. Postoperatively, the patient remained in the ICU for two days after the surgery; he developed a minor complication (surgical site infection (SSI) grade 1) and was later transferred to the ward and discharged after a week.

## Introduction

Hernias of the abdominal wall are protrusions of abdominal contents through weaknesses or defects in the abdominal wall. They are common entities, with an estimated 120,000 hernia operations performed annually in the United Kingdom, making this the most frequent surgical procedure nationwide [1,2]. Hernias are generally classified as external or internal. External hernias include indirect inguinal (the most common type, particularly in men, located within the inguinal canal) [3], direct inguinal (above the inguinal ligament), and femoral (below the inguinal ligament, more common in women) [4,5]. Other abdominal wall hernias include umbilical hernias (often in infants due to failure of closure of the umbilical ring), epigastric hernias (through small defects in the linea alba above the umbilicus) [6], incisional hernias (at previous surgical sites), and Spigelian hernias (through the linea semilunaris along the lateral border of the rectus abdominis) [7]. Internal hernias occur when abdominal contents protrude through peritoneal or mesenteric defects within the abdominal cavity.

Abdominal wall hernias are considered to be a frequent surgical pathology with an approximate prevalence of 1. 7% in all age groups [8]. Indirect inguinal hernias account for 75% of all inguinal hernias; hence, their repair is a common operation. Indirect inguinal hernias with sliding components account for 2-5% [9,10]. According to Robert Bendavid, sliding inguinal hernias can be classified into three types. The first type occurs when the wall of a viscus is included in the peritoneal sac. The second type involves both a retroperitoneal viscus and its mesentery forming part of the peritoneal sac. The third type is characterised by a minimal or absent peritoneal sac. Sliding inguinal hernias are most often identified intraoperatively, although they can also be detected using CT imaging [11,12].

Groin hernias, particularly inguinal hernias, develop in the groin region of the abdomen [13]. Females account for a small segment of patients with indirect inguinal hernias because of the process of development of the canal of Nuck, the female homologue of the processus vaginalis, which normally obliterates early in life and therefore rarely leaves a patent pathway for herniation [14]. Direct inguinal hernias are those where the abdominal contents come through a weakness at the level of the transversalis fascia within Hesselbach’s triangle, that is, the area of the groin. It usually develops later in life because of neutralisation or weakening of the muscles of the abdomen and increased intrabdominal pressure brought about by factors such as aging or straining with various activities, such as lifting weights or constant coughing.

Signs of an inguinal hernia include swelling, a lump or bulge in the groin area, pain or discomfort, a burning and aching sensation, swelling in the scrotum, and heaviness in the groin. Incarceration, where the hernia cannot be reduced or forced back into the abdominal cavity, can result in strangulation, intense pain, vomiting, and, in most cases, may call for emergency surgery [15]. This case report aimed to highlight the importance of timely and adequate surgical treatment of giant inguinoscrotal hernias, especially in elderly patients.

## Case presentation

**Figure 1:**
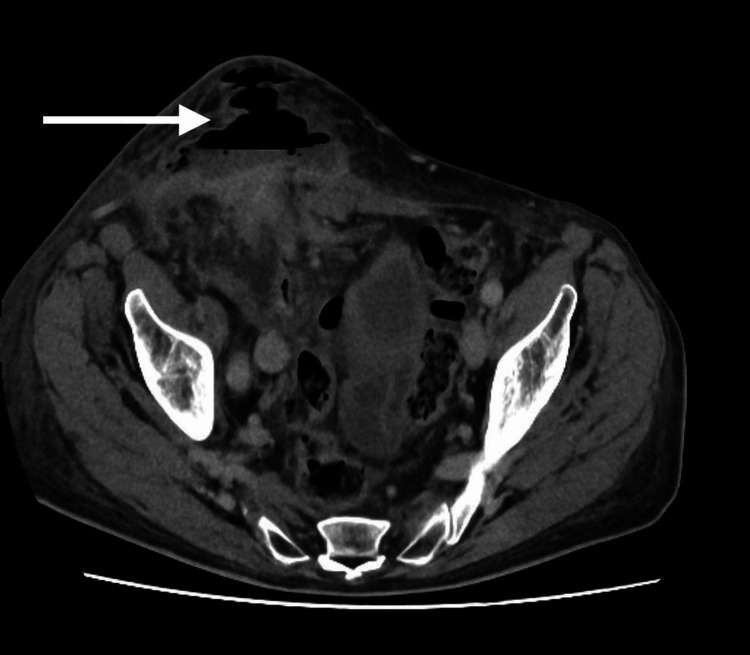
CTAP axial plane - 1 Collection suggestive of colon perforation CTAP: computed tomography of the abdomen and pelvis

**Figure 2:**
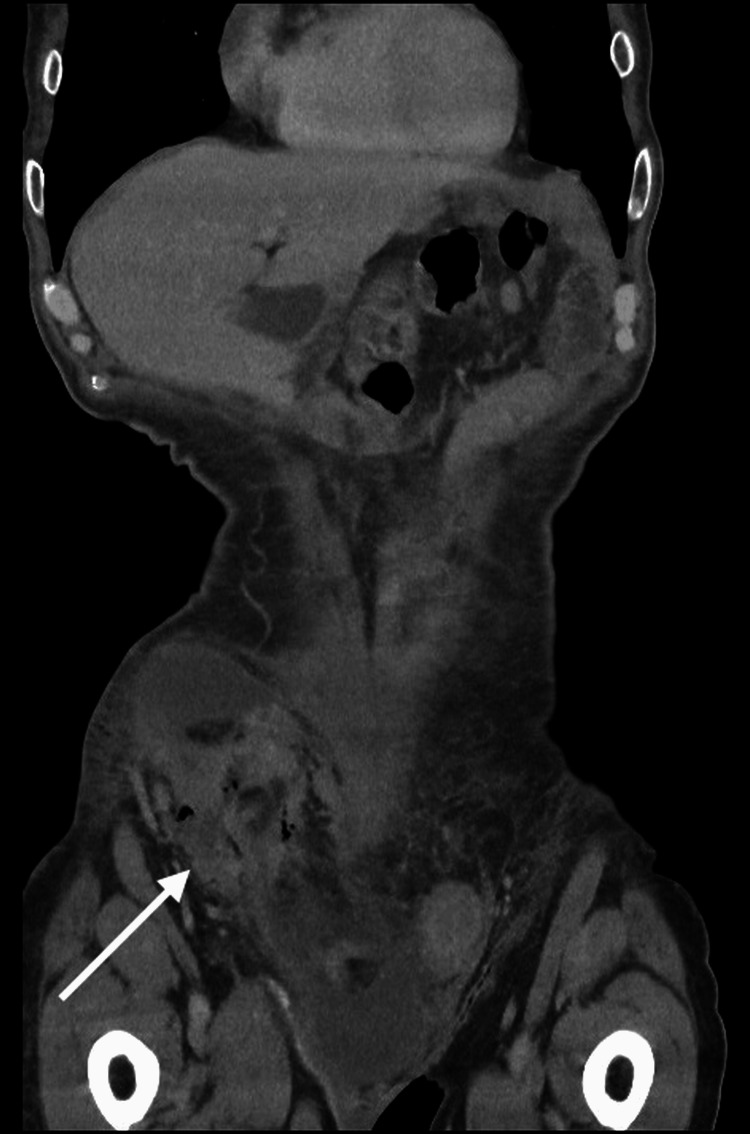
CTAP coronal plane - 1 Collection suggestive of colon perforation CTAP: computed tomography of the abdomen and pelvis

**Figure 3:**
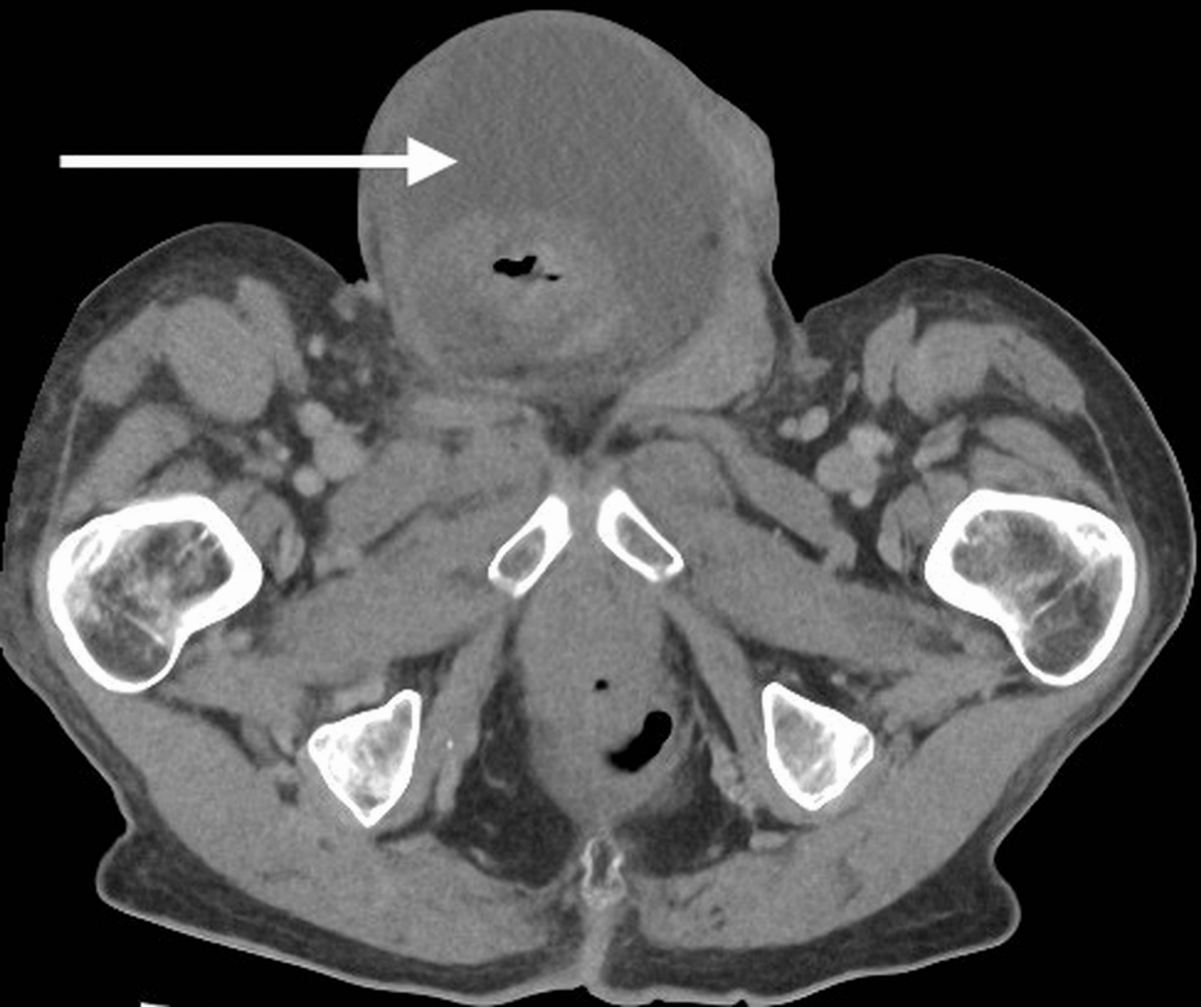
CTAP axial plane - 2 The hydrocele CTAP: computed tomography of the abdomen and pelvis

**Figure 4:**
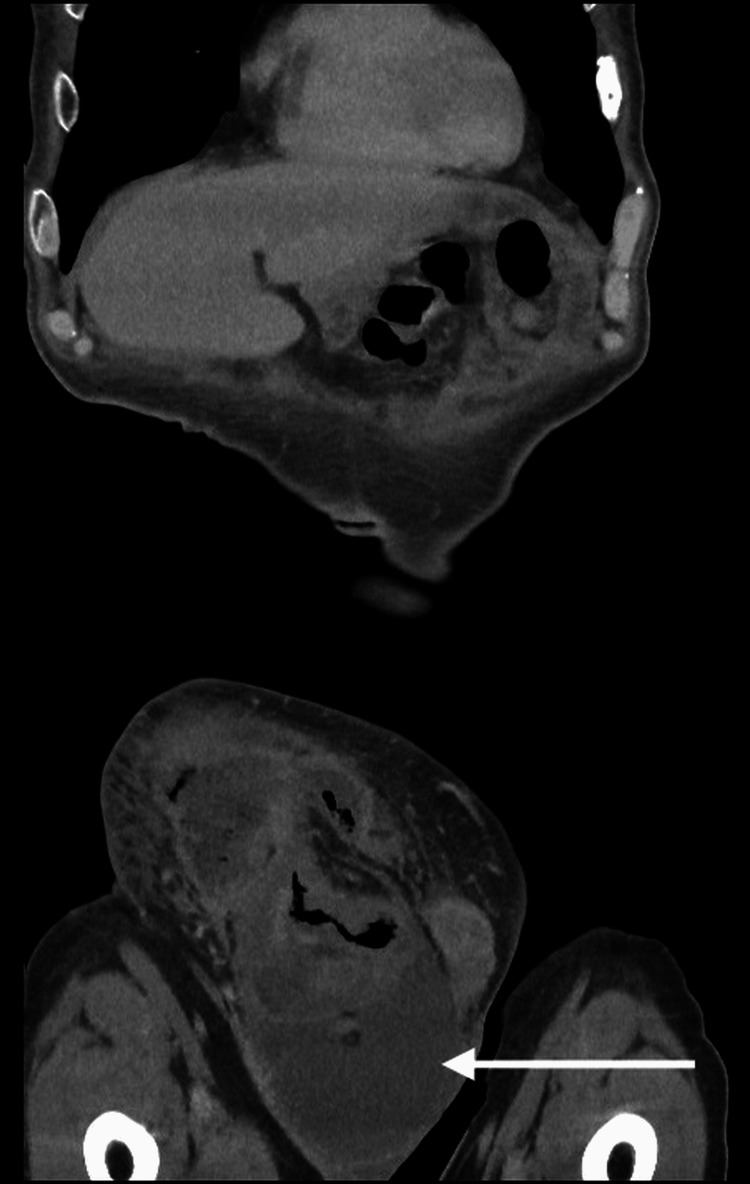
CTAP coronal plane - 2 The hydrocele CTAP: computed tomography of the abdomen and pelvis

A 75-year-old male was admitted through the surgical admissions unit, presenting with a large right-sided inguinoscrotal swelling for about three years. Over this time, it had become slightly larger but had not been causing any extreme pain. However, three days before the presentation, the patient had felt pain in the swollen area and noted some redness. The patient’s external physical examination revealed a large right-sided inguinoscrotal hernia, which was irreducible, tender, and had associated erythema. Based on the patient’s acute presentation and findings, an urgent CT of the abdomen and pelvis (CTAP) was performed, and this confirmed that the hernia contained the ascending colon and there was a collection suggestive of bowel perforation (Figures 1, 2) and hydrocele (Figures 3, 4).

An emergency laparotomy was performed via a midline abdominal incision. The intestines were examined, and there was a repair to an abdominal hernia. Operative findings were as follows: a large right-sided inguinoscrotal strangulated hernia containing the terminal ileum, caecum with a swollen appendix, ascending colon, omentum, and a seedling of the abdominal wall lateral to the hernia. Gangrene had caused a perforation, leading to fecal and gaseous contamination in the right groin and formation of an abscess. A significantly large hydrocele was observed.

The emergency surgery included a right hemicolectomy with the construction of an end ileostomy, drainage of the abscess, and hydrocelectomy. After surgery, the patient was kept in the ICU for two days and downgraded to the ward for another week. The recovery was complicated by a superficial incisional surgical site infection (grade 1), which was managed conservatively. The patient was subsequently discharged with follow-up arranged through a district nurse for wound care.

## Discussion

We described the case of a 75-year-old male who presented with a right-sided inguinoscrotal hernia complicated by bowel perforation and gangrene, highlighting the challenges in hernia management and the need for timely surgical evaluation. Inguinoscrotal hernias are relatively common, particularly in elderly patients. Their contents often include bowel loops, which may remain asymptomatic for long periods or cause only minimal symptoms. However, delayed treatment - often due to factors such as minimal early symptoms, comorbidities, or fear of surgery - results in an irreducible hernia that becomes incarcerated, then strangulated, ultimately leading to bowel ischemia and gangrene. Therefore, inguinoscrotal hernias are prone to complications such as strangulation and bowel ischemia, particularly in elderly patients if not surgically treated. According to Fitzgibbons et al., inguinal hernias are common in this age group [16]. 

Surgical intervention is specifically warranted in the case of a strangulated hernia. Mortality for the patients who required emergency surgical intervention for strangulated hernias was described by Lau et al. to be higher compared to elective surgery patients, due to the increased level of bowel ischemia and sepsis in the emergency setting [17]. In our case, the intraoperative findings were severe; however, the patient’s postoperative recovery demonstrates that timely and appropriate surgical intervention can reduce mortality and improve outcomes. Incarcerated and strangulated hernias complicated with abscesses can involve significant management difficulties. Wilson et al. emphasised adequate surgical access and removal of the infected tissues and foreign material to minimise the risk of postoperative infections in such cases. Our patient required massive surgical intervention, including bowel resection and abscess drainage, to control the grades of the complications encountered and aid in healing [18].

Simultaneous surgical correction of associated conditions such as hydrocele, along with hernia repair, eliminates the need for future procedures and reduces the risk of complications. Performing hydrocelectomy at the same time as hernia surgery simplifies postoperative care and minimises the chance of recurrence or persistence of scrotal swelling, as supported by the patient’s smooth postoperative course without complications [19]. A study by De Simone et al. and a retrospective analysis by Jensen et al. investigated the surgical outcomes of elderly patients who were affected by strangulated hernias. They emphasised that early diagnosis and surgical intervention can reduce mortality and morbidity rates significantly and prevent complications such as bowel ischemia and perforation [20].

Limitations

This report's findings are based on a single case, thereby limiting generalisability. It lacks comparative analysis with other cases, detailed follow-up data, and preoperative data, which could have provided a more comprehensive understanding of the hernia's complexity and informed the surgical approach.

## Conclusions

It is crucial to emphasise the necessity of early management of complicated hernias in geriatric patients to prevent adverse outcomes of the intervention. This report discussed a case of a 75-year-old male with a right inguinoscrotal hernia that caused bowel perforation and gangrenous condition, which highlights the severity of the condition and, consequently, the necessity of early treatment for the aforementioned pathology. The outcomes following right hemicolectomy, ileostomy, drain abscess, and hydrocelectomy proved that complex surgical approaches grounded on preliminary findings are effective and crucial. Prompt diagnosis and surgical intervention are essential for preventing complications like bowel perforation and gangrene. Advanced imaging techniques like CT scans can aid in accurate diagnosis and surgical planning. A multidisciplinary approach, including surgeons, radiologists, and intensivists, is essential for comprehensive care. Patient education about potential risks and regular follow-up is crucial for recovery and preventing recurrence.
